# Breed-by-Altitude Interaction Reveals Cardiac miRNA–mRNA Regulatory Hubs Associated with Hypoxia Adaptation in Tibetan Pigs

**DOI:** 10.3390/ijms27209246

**Published:** 2026-10-18

**Authors:** Pan Li, Wei Cheng, Peng Shang, Zhu Tao, Hao Zhang, Bo Zhang

**Affiliations:** 1 Frontiers Science Center for Molecular Design Breeding, China Agricultural University, Beijing 100193, China; lipan1011@cau.edu.cn (P.L.); chengwei@cau.edu.cn (W.C.); taozhu2020@cau.edu.cn (Z.T.); hzhang@cau.edu.cn (H.Z.); 2 National Engineering Laboratory for Animal Breeding, Beijing Key Laboratory for Animal Genetic Improvement, College of Animal Science and Technology, China Agricultural University, Beijing 100193, China; 3 Department of Animal Husbandry, Xizang Agricultural and Animal Husbandry University, Linzhi 860000, China; shangpeng1984@xza.edu.cn

**Keywords:** Tibetan pig, hypoxia adaptation, breed-by-altitude interaction, miRNA–mRNA network

## Abstract

MicroRNAs (miRNAs) are important post-transcriptional regulators of cardiac responses to hypoxia, but how breed and altitude interact to shape cardiac miRNA expression remains unclear. We used a 2 × 2 factorial design involving Tibetan and Yorkshire pigs raised at high (3000 m) or low (100 m) altitude. Small RNA sequencing identified 1104 miRNAs, including 370 known and 734 putative novel miRNA candidates. Pairwise comparisons revealed greater breed-related divergence at high altitude and a stronger altitude-associated miRNA response in Tibetan pigs than in Yorkshire pigs. The breed-by-altitude interaction analysis identified 94 breed-by-altitude interaction-responsive miRNAs, including 25 not detected by pairwise comparisons. K-means clustering further identified a Tibetan high-altitude-enriched miRNA module whose predicted targets were associated with hypoxia-inducible factor 1 (HIF-1), mechanistic target of rapamycin (mTOR), adrenergic, calcium, and metabolic pathways. Integration with previously published cardiac messenger RNA (mRNA) differential-expression results for high-altitude Tibetan versus Yorkshire pigs, generated from the corresponding biological samples, prioritized 319 anti-correlated miRNA–mRNA pairs involving 19 miRNAs and 224 mRNAs, with ssc-miR-671-5p as the leading network hub. Quantitative real-time polymerase chain reaction (qRT-PCR), correlation analysis, and miRanda prediction prioritized three candidate regulatory pairs: ssc-miR-671-5p–*ATP1B2*, novel-miR-212–*ADCY6*, and ssc-miR-296-3p–*PFKM*. These findings characterize breed-dependent, altitude-associated cardiac miRNA expression patterns and prioritize candidate miRNA–mRNA relationships for future functional investigation.

## 1. Introduction

Adaptation to high-altitude environments provides a powerful natural model for understanding how chronic hypobaric hypoxia shapes cardiovascular physiology in mammals. Reduced ambient oxygen availability imposes persistent constraints on oxygen delivery, vascular homeostasis, and myocardial energy metabolism, requiring the heart to maintain function under sustained hypoxic stress [1]. When these compensatory responses are inadequate, chronic altitude exposure can promote pulmonary vascular remodeling, pulmonary hypertension, and right ventricular overload, ultimately compromising cardiopulmonary performance [2,3]. Understanding the molecular basis of cardiac hypoxia tolerance is therefore important for both evolutionary biology and cardiovascular research.

The Tibetan pig (*Sus scrofa*), an indigenous breed of the Qinghai–Tibet Plateau, represents an informative livestock model for studying high-altitude adaptation. Previous studies have shown that Tibetan pigs display distinctive cardiovascular and hypoxia-related features, including altered vascular architecture in the heart, differential expression of hypoxia-responsive genes, and genomic signatures of long-term adaptation to plateau environments [4,5]. Broader work on high-altitude mammals has further highlighted the importance of genes in the hypoxia-inducible factor (HIF) pathway, particularly *EPAS1* and *EGLN1*, in shaping adaptive responses to chronic hypoxia [6,7]. However, although genomic and transcriptomic studies have identified candidate loci and expression differences in Tibetan pigs, the regulatory architecture that connects genetic background to cardiac molecular plasticity remains incompletely understood. MicroRNAs (miRNAs) are small non-coding RNAs that regulate gene expression post-transcriptionally, primarily through messenger RNA (mRNA) degradation or translational repression [8,9]. In the cardiovascular system, miRNAs have been implicated in multiple stress-responsive processes, including mitochondrial metabolism, apoptosis, angiogenesis, and pathological remodeling [10,11]. Under hypoxic conditions in particular, several miRNAs have been shown to participate in cell survival and metabolic adaptation, suggesting that miRNA-mediated regulation may be an important component of cardiac tolerance to oxygen deprivation [12,13]. Nevertheless, the contribution of miRNA regulation to high-altitude adaptation in livestock species remains insufficiently resolved.

A major limitation of previous studies is that they have largely relied on simple pairwise comparisons, such as breed contrasts under one environment or altitude contrasts within one population. In Tibetan pigs, for example, earlier cardiac miRNA profiling mainly compared Tibetan and Yorkshire pigs under high-altitude conditions, which was informative for differential expression but could not explicitly distinguish constitutive breed differences from environment-dependent regulatory responses [14]. From a quantitative genetics perspective, such context dependence is broadly related to genotype-by-environment interaction, in which different genetic backgrounds show non-parallel responses across environments. In the present population-based design, however, the statistical effect tested was specifically the breed-by-altitude interaction rather than an individual genotype-by-environment effect. Applying a factorial interaction framework to cardiac miRNA expression may therefore reveal breed-dependent altitude-associated patterns that are not detectable through conventional pairwise analyses alone [15]. In the present study, we used a 2 × 2 factorial design involving Tibetan and Yorkshire pigs raised at high altitude (3000 m) and low altitude (100 m) to characterize the cardiac miRNA landscape associated with breed, altitude, and breed-by-altitude interaction. By combining small RNA sequencing with generalized linear modeling and integrative analysis of a previously published cardiac transcriptomic dataset comparing Tibetan and Yorkshire pigs at high altitude, we aimed to identify breed-by-altitude interaction-responsive miRNAs and prioritize candidate miRNA–mRNA relationships associated with the Tibetan high-altitude cardiac response. Our study provides a post-transcriptional perspective on how breed background and rearing altitude jointly shape cardiac molecular variation in a high-altitude-adapted pig breed.

## 2. Results

### 2.1. Identification and Expression Characteristics of miRNAs

**Figure 1:**
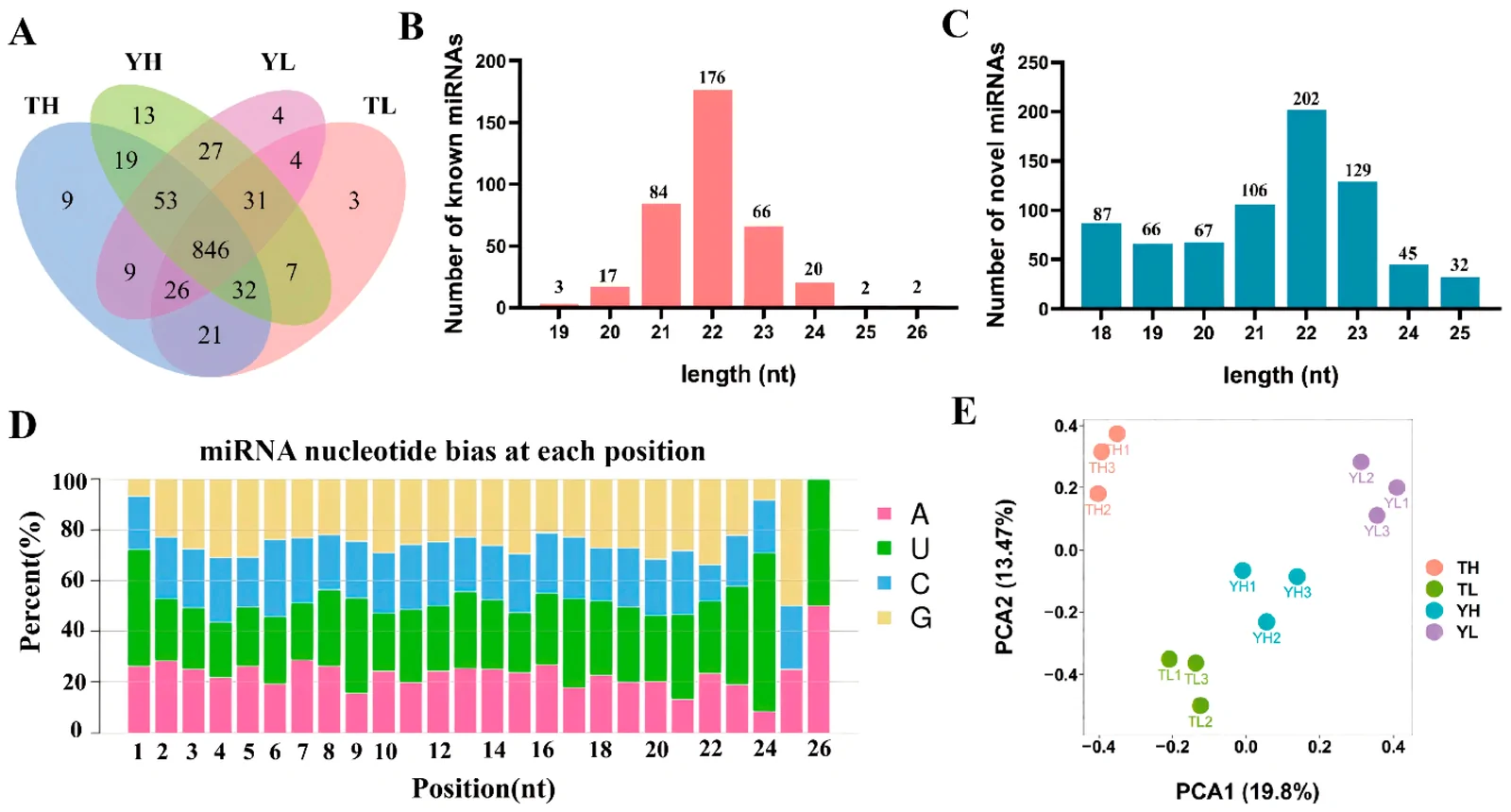
Identification and expression characteristics of microRNAs (miRNAs) in pig heart tissue. (**A**) Venn diagram showing the overlap and unique miRNAs among the four groups: Tibetan pigs at high altitude (TH), Tibetan pigs at low altitude (TL), Yorkshire pigs at high altitude (YH), and Yorkshire pigs at low altitude (YL). (**B**) Length distribution of known miRNAs. (**C**) Length distribution of putative novel miRNA candidates. (**D**) Nucleotide bias at each position of miRNAs. (**E**) Principal component analysis (PCA) of miRNA expression profiles across all samples, illustrating the clustering pattern among different groups.

To investigate the miRNA expression landscape in pig heart tissue, small RNA sequencing was performed on twelve samples from four experimental groups: Tibetan pigs at high altitude (TH), Tibetan pigs at low altitude (TL), Yorkshire pigs at high altitude (YH), and Yorkshire pigs at low altitude (YL). After removing low-quality reads and adaptor sequences, a total of 144.39 million clean reads were obtained from all libraries. The sequencing quality of all samples was high, with Q30 values exceeding 94.92%. The clean reads were mapped to the pig reference genome, yielding mapping rates ranging from 64.96% to 78.49% (Table S2), indicating that the sequencing data were of high quality and suitable for subsequent analyses. Based on the sequencing results, a total of 1104 miRNAs were identified across all samples, including 370 known miRNAs and 734 putative novel miRNA candidates. Among the four groups, 1016 miRNAs were detected in TH, 1029 in YH, 971 in TL, and 1001 in YL (Table S3). Venn diagram analysis further revealed the overlap and unique miRNAs among the four groups. Specifically, 9, 13, 4, and 3 group-specific miRNAs were uniquely detected in TH, YH, YL, and TL, respectively (Figure 1A). Length distribution analysis showed that most known miRNAs ranged from 19 to 26 nt, while putative novel miRNAs mainly ranged from 18 to 25 nt, with 22 nt being the most abundant length, consistent with the typical characteristics of mature miRNAs (Figure 1B,C). Nucleotide bias analysis at each position demonstrated a strong preference for U at the 5′ end (Figure 1D), consistent with Dicer cleavage specificity and indicative of high data quality. Principal component analysis (PCA) based on miRNA expression profiles revealed clear differences among the four groups. Notably, the TH group showed distinct separation from the other groups, whereas YH, TL, and YL exhibited relatively similar expression patterns (Figure 1E), suggesting potential differences in miRNA regulatory mechanisms associated with altitude adaptation and breed differences.

### 2.2. Differentially Expressed (DE) miRNAs Under Breed and Altitude Contrasts

**Figure 2:**
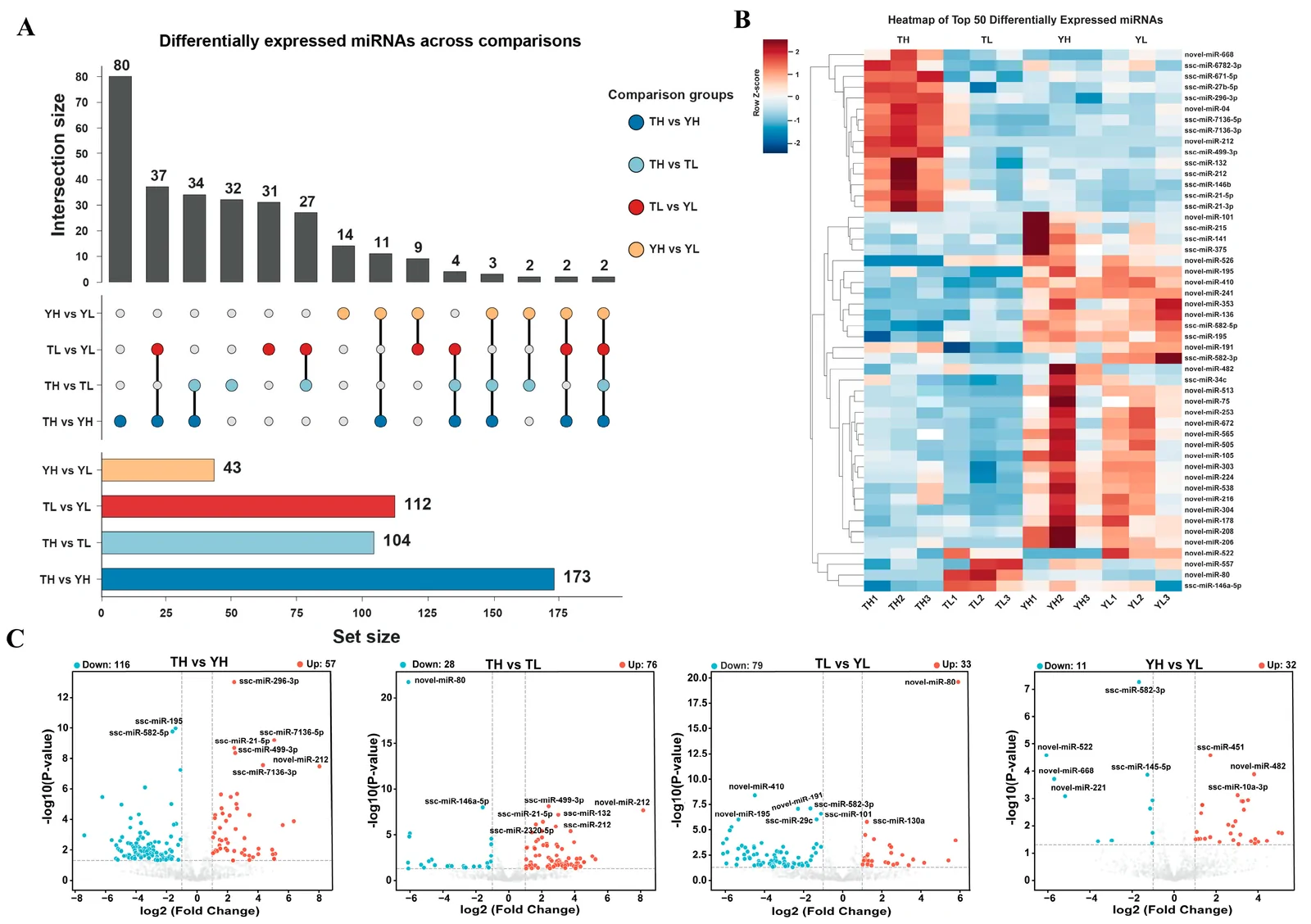
Differentially expressed (DE) miRNAs across breed and altitude contrasts. (**A**) UpSet plot showing differential miRNAs expression among the four pairwise comparisons. Vertical bars indicate intersection sizes, whereas horizontal bars indicate the total number of DE miRNAs in each comparison. (**B**) Hierarchical clustering heatmap of the top 50 DE miRNAs across the 12 samples. (**C**) Volcano plots of miRNAs differential expression across the four pairwise comparisons. Red and blue dots indicate upregulated and downregulated candidate miRNAs (|log_2_ Fold Change| ≥ 1 and nominal *p* < 0.05), respectively; gray dots indicate miRNAs not meeting these criteria. Dashed lines indicate the corresponding screening thresholds. Representative miRNAs are labeled.

To characterize cardiac miRNA expression differences associated with breed and altitude, we examined DE miRNAs across the four experimental contrasts. Pairwise analysis identified 173, 104, 112, and 43 candidate DE miRNAs in the TH vs. YH, TH vs. TL, TL vs. YL, and YH vs. YL comparisons, respectively (Figure 2A, Table S4). After merging the four lists and removing overlapping miRNAs, a total of 288 unique DE miRNAs were identified. Notably, more breed-associated DE miRNAs were detected at high altitude (173 in TH vs. YH) than at low altitude (112 in TL vs. YL), suggesting that breed-related miRNA differences may be more pronounced under high-altitude conditions. Furthermore, more altitude-associated DE miRNAs were identified in Tibetan pigs (104 in TH vs. TL) than in Yorkshire pigs (43 in YH vs. YL), suggesting a broader altitude-associated miRNA response in Tibetan pigs. To compare their expression patterns, hierarchical clustering was performed using the top 50 DE miRNAs. The heatmap revealed distinct expression blocks among the four groups, with the TH samples showing a particularly distinct miRNA expression profile (Figure 2B). The UpSet analysis further showed that 80 DE miRNAs occurred only in the TH vs. YH comparison, indicating that many breed-associated differences were specific to the high-altitude comparison (Figure 2A). In contrast, 37 DE miRNAs were shared between the two breed contrasts, representing a subset of breed-associated expression differences observed at both altitudes. Volcano plots further illustrated the direction, fold-change magnitude, and statistical significance of miRNA expression differences in each contrast (Figure 2C). Among the four comparisons, TH vs. YH contained the largest number of DE miRNAs. Representative DE miRNAs included ssc-miR-296-3p in the TH vs. YH contrast and novel-miR-80 in the TH vs. TL contrast. Together, these pairwise comparisons revealed contrast-specific miRNA expression patterns and motivated formal testing of the breed-by-altitude interaction.

### 2.3. Breed-by-Altitude Interaction Shapes Cardiac miRNA Expression Patterns

**Figure 3:**
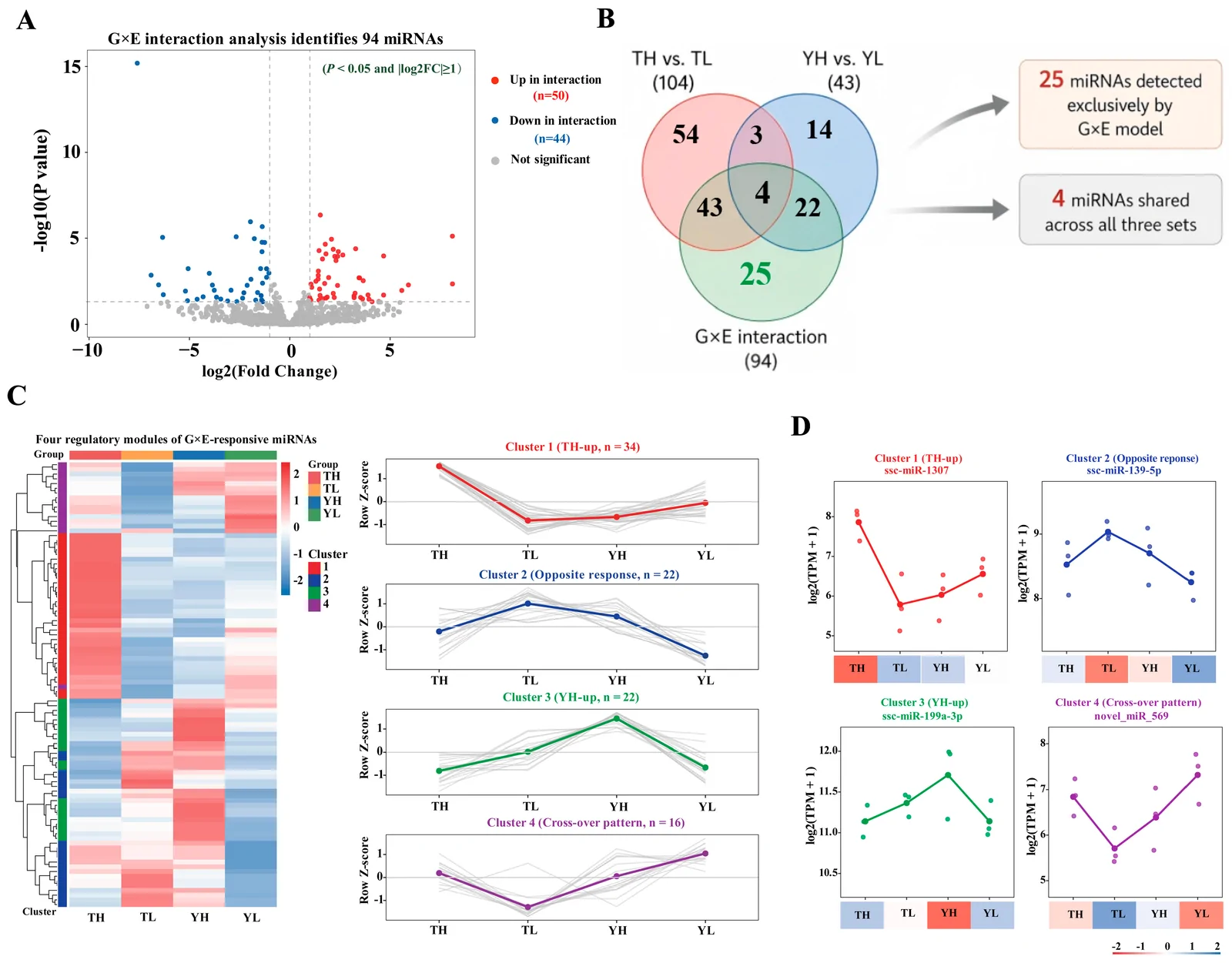
Breed-by-altitude interaction shapes cardiac miRNA expression patterns. (**A**) Volcano plot of breed-by-altitude interaction effects. Red and blue dots indicate candidate miRNAs with positive and negative interaction coefficients, respectively (|log_2_ Fold Change| ≥ 1; nominal *p* < 0.05). Gray dots indicate other miRNAs, and dashed lines mark the screening thresholds. (**B**) Venn diagram showing the overlap between interaction-responsive miRNAs and DE miRNAs identified in the TH vs. TL and YH vs. YL contrasts. (**C**) Heatmap and k-means clustering of 94 candidate interaction-responsive miRNAs into four clusters. Gray lines represent individual miRNA expression profiles, whereas colored lines indicate cluster means. (**D**) Expression profiles of representative miRNAs from each cluster. Upper panels show log_2_(transcripts per million [TPM] + 1) expression across the TH, TL, YH, and YL groups, whereas lower panels show the corresponding 1 × 4 heatmaps of group-mean expression.

We next examined whether altitude-associated cardiac miRNA expression differed between Tibetan and Yorkshire pigs through breed-by-altitude interaction analysis. By jointly modeling breed, altitude, and their interaction, we identified 94 candidate interaction-responsive miRNAs using the exploratory threshold of |log_2_ Fold Change| ≥ 1 and nominal *p* < 0.05 (Figure 3A, Table S5). These results showed that altitude-associated miRNA expression differed according to breed background. Among these, 50 miRNAs had positive interaction coefficients and 44 had negative coefficients, indicating non-parallel altitude-associated expression differences in both directions. To compare the interaction analysis with conventional pairwise screening, the 94 interaction-responsive miRNAs were compared with DE miRNAs identified in the TH vs. TL and YH vs. YL contrasts. Although many interaction-responsive miRNAs overlapped with one or both altitude-responsive sets, 25 were absent from both within-breed DE lists, whereas four were shared among all three sets (Figure 3B). These results demonstrate that interaction analysis identified breed-dependent altitude-associated expression patterns that were not recovered by pairwise screening alone. To characterize their expression patterns, the 94 interaction-responsive miRNAs were partitioned into four clusters using k-means clustering (Figure 3C). Cluster 1 (n = 34) corresponded to a TH-up pattern, with elevated expression in Tibetan pigs under high-altitude conditions relative to the other groups. Cluster 2 (n = 22) exhibited an opposite response pattern, in which altitude-associated expression changes differed in direction between Tibetan and Yorkshire pigs. Cluster 3 (n = 22) showed a YH-up pattern, with elevated expression in Yorkshire pigs under high-altitude conditions. Cluster 4 (n = 16) displayed a cross-over pattern, characterized by a non-parallel trajectory across the four groups. Together, these clustered patterns revealed distinct altitude-associated miRNA expression trajectories between Tibetan and Yorkshire pigs. Representative miRNAs from each cluster illustrated the corresponding interaction-responsive expression trajectories (Figure 3D). Specifically, ssc-miR-1307 represented the TH-up pattern of Cluster 1, whereas ssc-miR-139-5p exemplified the opposite response pattern of Cluster 2. In addition, ssc-miR-199a-3p and novel-miR-569 recapitulated the dominant expression trends of Clusters 3 and 4, respectively. Based on its TH-enriched expression pattern, Cluster 1 was prioritized as a Tibetan high-altitude-associated miRNA cluster for subsequent target prediction and integrated network analysis.

### 2.4. Functional Annotation of Predicted Target Genes of Differentially Expressed and Interaction-Responsive miRNAs

**Figure 4:**
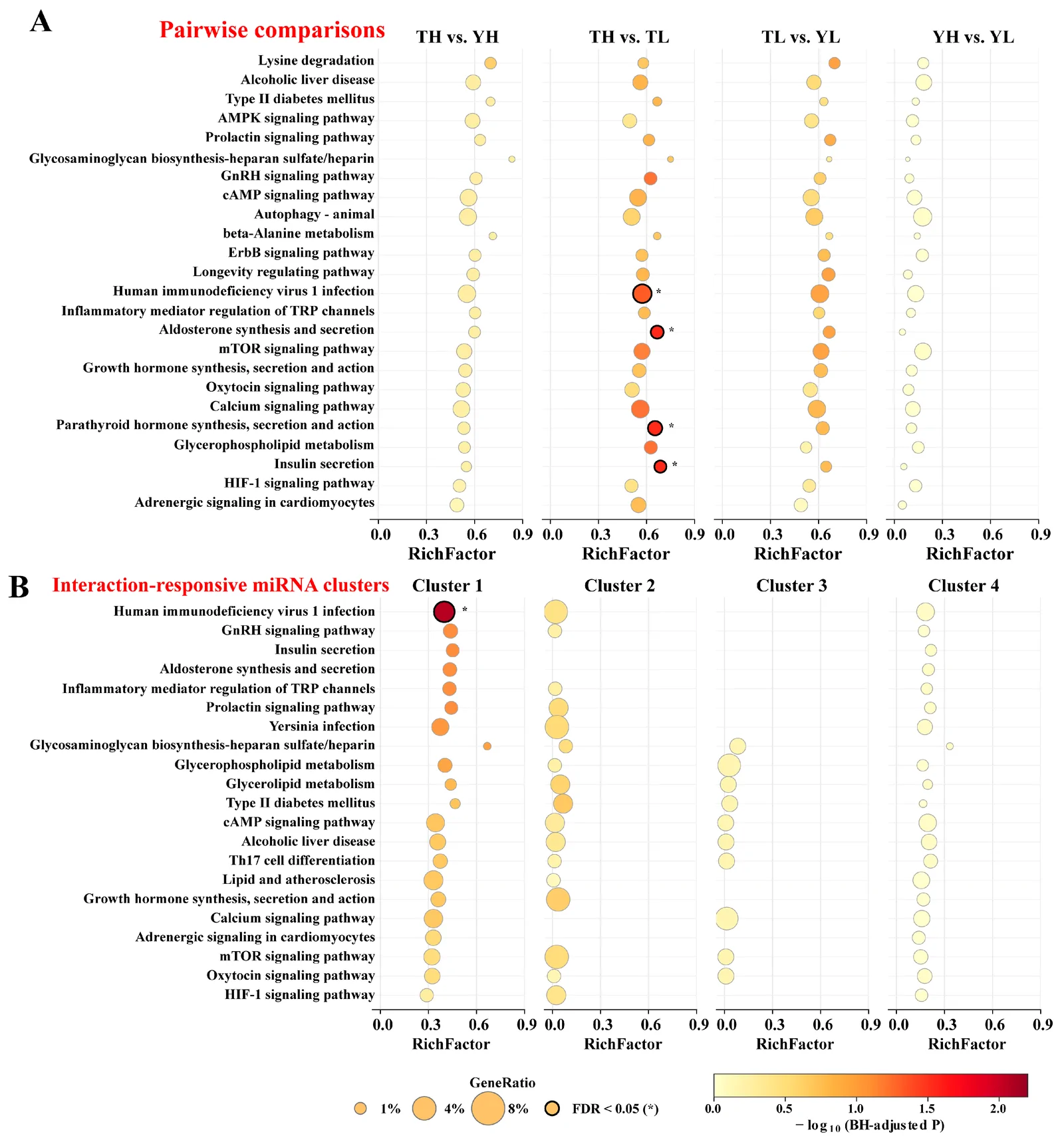
Kyoto Encyclopedia of Genes and Genomes (KEGG) pathway enrichment analysis of predicted target genes of differentially expressed and interaction-responsive miRNAs. (**A**) Bubble plots for four pairwise comparisons (TH vs. YH, TH vs. TL, TL vs. YL, and YH vs. YL) in heart tissue. (**B**) Bubble plots for four interaction-responsive miRNA clusters. Within each panel, the same selected pathways are shown across all four analyses. The horizontal position represents RichFactor, bubble area represents GeneRatio, and color represents −log_10_ of the Benjamini–Hochberg-adjusted *p* value. Black outlines and asterisks indicate adjusted *p* < 0.05.

To explore the potential biological functions associated with cardiac miRNA differences, Kyoto Encyclopedia of Genes and Genomes (KEGG) pathway enrichment analysis was performed for the predicted targets of differentially expressed (DE) and interaction-responsive miRNAs using genes expressed across the 12 cardiac samples as the background. The pairwise target sets showed different pathway-ranking profiles. Four pathways reached the Benjamini–Hochberg-adjusted significance threshold in the TH vs. TL comparison, whereas no pathway reached this threshold in the other three pairwise comparisons (Figure 4A, Table S6). Cluster-wise analysis was subsequently performed for the four expression-pattern clusters of interaction-responsive miRNAs (Figure 4B, Table S6). Cluster 1 represented a TH-enriched expression pattern, with higher miRNA abundance in Tibetan pigs raised at high altitude than in the other groups. One KEGG term reached adjusted *p* < 0.05 in Cluster 1, whereas no pathway reached the adjusted significance threshold in Clusters 2–4 (Figure 4B and Table S6). The predicted target set of Cluster 1 contained genes annotated to several pathways of potential relevance to cardiac hypoxia responses, including hypoxia-inducible factor 1 (HIF-1), mechanistic target of rapamycin (mTOR), calcium, and adrenergic signaling in cardiomyocytes. Calcium, mTOR, and adrenergic signaling showed nominal enrichment, although none met the Benjamini–Hochberg-adjusted significance threshold. Gonadotropin-releasing hormone (GnRH), oxytocin, and growth hormone signaling likewise did not meet the adjusted significance threshold and were therefore considered exploratory signals. Based on its TH-enriched expression pattern and the potential functional relevance of its predicted targets, Cluster 1 was prioritized for subsequent integrative miRNA–mRNA analysis.

### 2.5. Integrated miRNA–mRNA Analysis Prioritizes Candidate Regulatory Relationships Associated with the Tibetan High-Altitude Response

**Figure 5:**
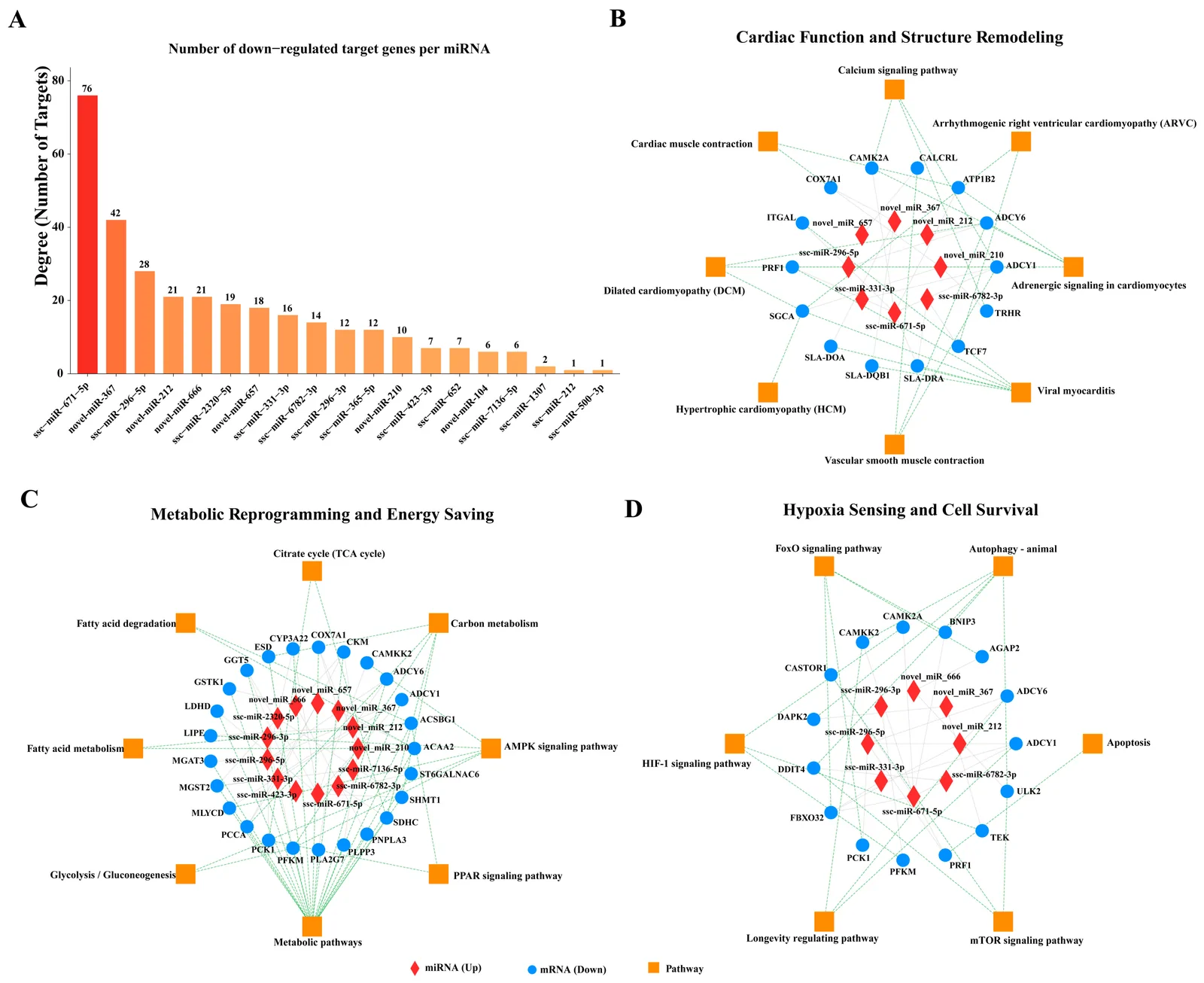
Integrated analysis of candidate miRNA–mRNA relationships associated with breed-by-altitude interaction. (**A**) Node-degree ranking of the 19 Cluster 1 interaction-responsive miRNAs within the candidate network. (**B**–**D**) miRNA–mRNA–pathway networks organized into functional categories related to cardiac function and structural remodeling (**B**), metabolic regulation (**C**), and hypoxia sensing and cell survival (**D**). Red diamonds represent miRNAs, blue circles represent candidate mRNA partners, and orange squares represent Kyoto Encyclopedia of Genes and Genomes (KEGG) pathways. Gray solid lines indicate candidate miRNA–mRNA pairs retained after target prediction and correlation filtering, whereas green dashed lines indicate mRNA–pathway annotations.

To identify candidate miRNA–mRNA relationships associated with breed-by-altitude interaction, we integrated Cluster 1 miRNAs with differentially expressed mRNAs identified in the previously published TH vs. YH cardiac transcriptomic comparison. Integration of TH-enriched Cluster 1 miRNAs with downregulated mRNAs in the TH vs. YH contrast identified 319 candidate anti-correlated miRNA–mRNA pairs involving 19 miRNAs and 224 mRNAs. Node-degree analysis revealed substantial differences in connectivity among the 19 miRNAs. ssc-miR-671-5p had the highest node degree, with 76 candidate mRNA connections, followed by novel-miR-367 with 42 and ssc-miR-296-5p with 28 (Figure 5A, Table S7). The major hub miRNAs showed relatively high connectivity within the prediction- and correlation-filtered candidate miRNA–mRNA network. For functional interpretation, candidate pairs were organized into three categories according to the KEGG pathway annotations of their associated mRNAs. The cardiac function and structural-remodeling category included the candidate ssc-miR-671-5p–*ATP1B2* pair (Figure 5B). ssc-miR-671-5p was also connected to predicted targets annotated to adrenergic signaling in cardiomyocytes and calcium signaling. The metabolic category contained candidate targets annotated to oxidative phosphorylation, fatty acid metabolism, and other energy-related pathways (Figure 5C). Within this category, novel-miR-212–*ADCY6* was prioritized for subsequent evaluation, with *ADCY6* annotated to cyclic adenosine monophosphate (cAMP) signaling and adrenergic signaling in cardiomyocytes. Finally, the hypoxia-sensing and cell-survival category contained candidate targets annotated to the HIF-1, mTOR, and forkhead box O (FoxO) signaling pathways (Figure 5D). Candidate mRNAs in this category included *BNIP3*, *PFKM*, and *TEK*, which were associated with stress-response and metabolic pathways. Among these relationships, ssc-miR-296-3p–*PFKM* was selected for subsequent expression validation and correlation analysis.

### 2.6. Expression Validation and Evaluation of Candidate miRNA–mRNA Pairs

**Figure 6:**
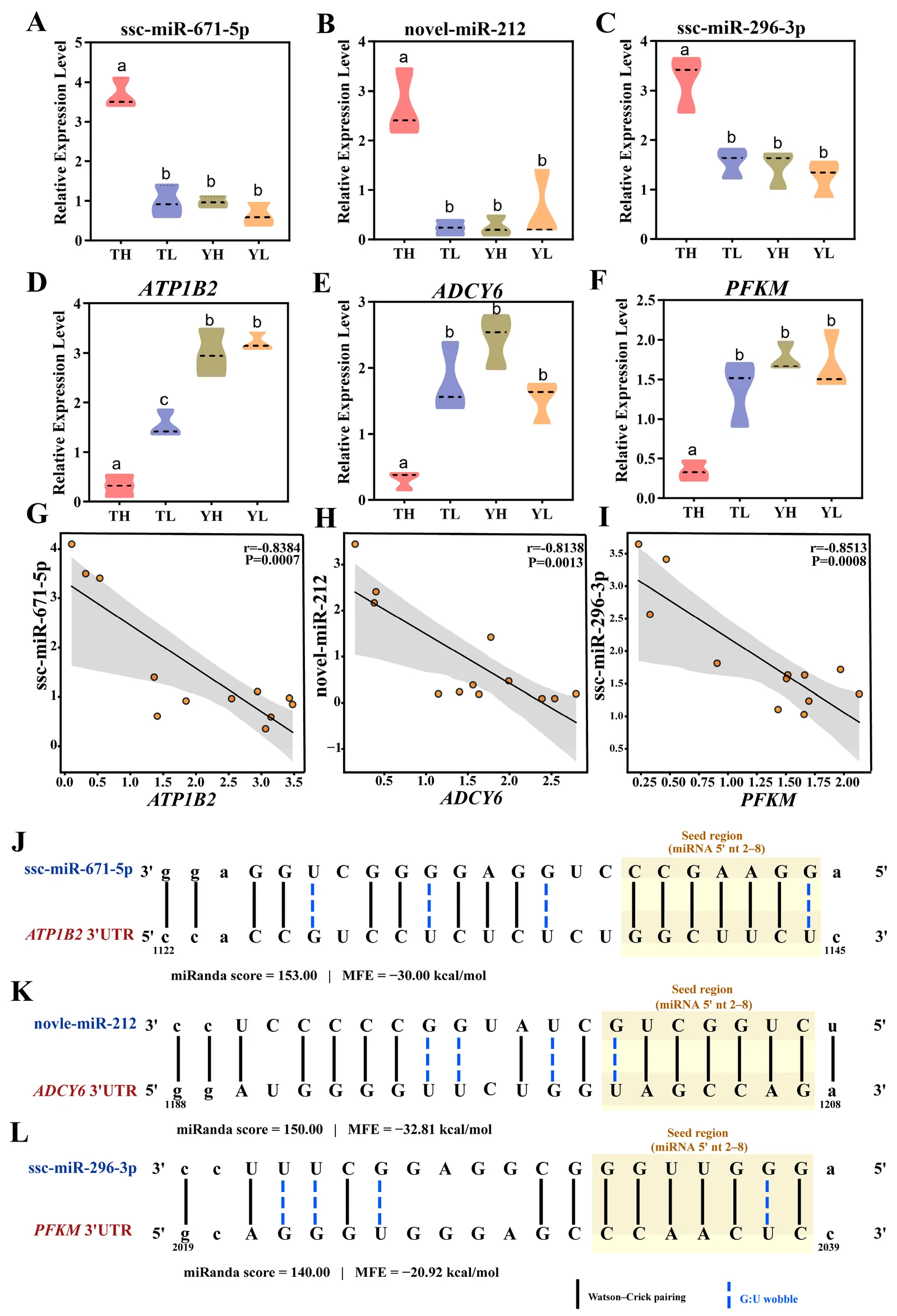
Expression and computational evaluation of prioritized candidate miRNA–mRNA pairs. (**A**–**F**) Relative expression measured by quantitative real-time polymerase chain reaction (qRT-PCR) in the same 12 biological samples used for sequencing. Violin plots show the relative expression of the prioritized miRNAs (**A**–**C**) and their candidate mRNA partners (**D**–**F**) across the TH, TL, YH, and YL groups. Different letters indicate significant differences among groups (*p* < 0.05; two-way analysis of variance (ANOVA) followed by Tukey’s multiple-comparison test). (**G**–**I**) Pearson correlations between miRNA and mRNA expression across all 12 samples. Orange dots represent individual biological samples, and shaded bands indicate 95% confidence intervals. Pearson correlation coefficients (r) and unadjusted *p* values are shown. (**J**–**L**) miRanda-predicted miRNA–3′ untranslated region (3′ UTR) duplexes. Yellow shading indicates miRNA seed regions; solid black and dashed blue lines represent Watson–Crick and G–U wobble pairs, respectively.

To examine the expression patterns of the prioritized miRNA–mRNA pairs, we evaluated ssc-miR-671-5p–*ATP1B2*, novel-miR-212–*ADCY6*, and ssc-miR-296-3p–*PFKM* using quantitative real-time polymerase chain reaction (qRT-PCR), correlation analysis, and miRanda prediction. These pairs were selected based on their expression patterns, network connectivity, and pathway annotations related to cardiac function and hypoxia responses. The qRT-PCR expression profiles across the four groups were consistent with the TH-enriched pattern observed for Cluster 1 in the small RNA sequencing analysis. All three candidate miRNAs showed their highest relative expression in the TH group and comparatively lower expression in the other groups (Figure 6A–C). Their corresponding candidate mRNAs, *ATP1B2*, *ADCY6*, and *PFKM*, showed their lowest relative abundance in TH hearts, resulting in inverse group-level expression patterns (Figure 6D–F). Pearson correlation analysis across the 12 biological replicates showed negative correlations for all three candidate pairs: ssc-miR-671-5p–*ATP1B2* (r = −0.8384, *p* = 0.0007), novel-miR-212–*ADCY6* (r = −0.8138, *p* = 0.0013), and ssc-miR-296-3p–*PFKM* (r = −0.8513, *p* = 0.0008) (Figure 6G–I). Potential binding sites for the three candidate pairs were further evaluated using miRanda. The predicted duplexes contained complementary pairing between the miRNA seed regions (nt 2–8) and putative binding sites in the corresponding mRNA mRNA 3′ untranslated regions (3′ UTRs) (Figure 6J–L). The low minimum free energy (MFE) values (−32.81 to −20.92 kcal/mol) and high miRanda scores further supported the plausibility of direct miRNA–mRNA recognition. Together, the concordant qRT-PCR expression patterns, negative correlations, and predicted binding sites supported the prioritization of these three candidate miRNA–mRNA pairs for future mechanistic testing.

## 3. Discussion

The present study indicates that cardiac miRNA expression in pigs is associated not only with breed and altitude, but also with their interaction. Using a 2 × 2 factorial design, we detected more breed-associated candidate miRNAs at high altitude than at low altitude. Meanwhile, more altitude-associated candidates were identified in Tibetan pigs than in Yorkshire pigs. The generalized linear model further identified 94 candidate breed-by-altitude interaction-responsive miRNAs, including 25 that were not captured by the two within-breed altitude comparisons. Together with the TH-enriched Cluster 1 module and the integrated miRNA–mRNA network, these findings indicate that the Tibetan high-altitude cardiac response is associated with context-dependent post-transcriptional variation rather than a uniform response across breeds. This interpretation is consistent with, while extending, previous studies of Tibetan pig adaptation. Earlier genomic and transcriptomic analyses have shown that Tibetan pigs possess distinctive cardiovascular and hypoxia-related features, including altered vascular architecture, differential expression of hypoxia-responsive genes, and genomic signatures of plateau adaptation [4,5]. Likewise, previous cardiac miRNA profiling in Tibetan pigs indicated that miRNA regulation may participate in hypoxia adaptation [14], but those analyses were largely based on direct pairwise comparisons under a single environmental context. In contrast, our factorial design enabled separate estimation of breed-, altitude-, and breed-by-altitude interaction-associated expression patterns. Rather than merely extending conventional differential-expression analysis, the breed-by-altitude interaction-responsive miRNAs identified here define a layer of regulatory variation in which cardiac miRNA trajectories depend on the combination of breed background and rearing altitude. More generally, genotype-by-environment interaction can reshape phenotypic and transcriptional responses across environments [15,16]. In the present study, this concept was evaluated operationally as a population-level breed-by-altitude interaction rather than an individual genotype-by-environment effect.

Maintaining myocardial function during chronic hypoxia involves adjustments in oxygen sensing, energy use, and contractile activity [17,18,19]. In this context, the predicted target annotations of TH-enriched Cluster 1 miRNAs suggest possible links to HIF-1, mTOR, calcium, adrenergic, and glycerophospholipid pathways. Although several of these pathways showed nominal enrichment, none met the Benjamini–Hochberg-adjusted significance threshold using cardiac-expressed genes as the background. The GnRH, oxytocin, and growth hormone terms also did not meet this threshold and may reflect shared signaling components or uncertainty in computational target prediction rather than specific neuroendocrine regulation. Previous studies have described metabolic and cardiovascular responses to cardiac hypoxia or high-altitude exposure [20,21,22], providing biological context for these observations. The Cluster 1 annotations therefore identify pathways for further investigation, but do not establish pathway activation or an integrated cardiometabolic adaptation program. Direct tests of miRNA–target interactions and pathway activity are needed to determine whether these candidates contribute to cardiac responses across contrasting altitude environments.

The integrated miRNA–mRNA analysis further identified candidate molecular relationships associated with the TH expression pattern. Among the 319 anti-correlated pairs in the filtered network, ssc-miR-671-5p showed the highest node degree, indicating relatively high connectivity within the prediction- and correlation-based candidate network. The candidate ssc-miR-671-5p–*ATP1B2* pair is of particular interest because *ATP1B2* is a component of the Na^+^/K^+^-ATPase complex, which is essential for maintaining transmembrane ion gradients and myocardial excitability [23,24]. Similarly, the candidate novel-miR-212–*ADCY6* pair links a predicted miRNA–mRNA regulation to cAMP-dependent adrenergic signaling, which plays an important role in cardiac contractility and remodeling [25]. The candidate ssc-miR-296-3p–*PFKM* pair is also biologically relevant because PFKM contributes to glycolytic metabolism, a process closely associated with myocardial adaptation to limited oxygen availability [18,26]. Notably, these three prioritized axes converge on complementary aspects of myocardial adaptation, linking post-transcriptional regulation to ion homeostasis (*ATP1B2*), cAMP-dependent signaling (*ADCY6*), and glycolytic remodeling (*PFKM*). This functional complementarity strengthens the biological plausibility of the network because it connects the major adaptive tasks of the hypoxic heart—electrophysiological stability, signal transduction, and metabolic flexibility—to specific candidate miRNA–mRNA relationships. Recent reviews of Na^+^/K^+^-ATPase biology, adenylyl cyclase isoforms 5 and 6 (AC5/AC6) signaling, and cardiac cAMP nanodomains further support the idea that these pathways occupy central positions in myocardial adaptation and remodeling [27,28,29,30]. Although the current data do not establish direct causality, the convergence of network topology, reciprocal expression patterns, negative correlation, and favorable binding energetics supports the biological plausibility of these axes as candidate mediators of adaptive remodeling in the Tibetan pig heart.

An important biological implication of our findings is that high-altitude adaptation in Tibetan pigs may involve a shift toward a more economical regulatory state under chronic hypoxia. Rather than simply increasing the expression of hypoxia-related genes, the adapted heart appears to employ miRNA-mediated fine-tuning of ion transport, adrenergic responsiveness, and metabolic pathways. Such a strategy could help preserve myocardial homeostasis by preventing excessive excitation, limiting maladaptive signaling, and optimizing energy utilization under reduced oxygen availability. This interpretation is in line with studies showing that chronically hypoxic hearts often rely on coordinated adjustments in mitochondrial function, substrate preference, and stress-response signaling to maintain performance [8,18,19]. In this framework, the interaction-responsive miRNA program identified here may function as a post-transcriptional buffering system that stabilizes myocardial physiology when environmental oxygen supply becomes limiting. This view is also compatible with the broader concept of hypoxamiRs, in which hypoxia-regulated miRNAs act not merely as downstream consequences of HIF activation, but as integrative regulators that couple oxygen sensing to tissue remodeling, survival, inflammation, and metabolic adaptation [31,32]. While this study identifies breed-dependent cardiac miRNA patterns across contrasting altitudes, altitude was confounded with study site. Thus, altitude-associated differences and breed-by-altitude interactions may also reflect other site-related factors and cannot be attributed exclusively to hypobaric hypoxia. The relatively modest sample size should also be considered when interpreting weaker interaction effects. The novel miRNA candidates remain computational predictions, and qRT-PCR validation of candidate novel-miR-212 did not confirm canonical miRNA biogenesis. Larger cohorts and functional studies are needed to validate these findings and clarify their roles in myocardial hypoxia tolerance. Importantly, our findings establish a framework for identifying context-dependent miRNA regulation associated with cardiac responses to chronic hypoxia and prioritize a focused set of candidate miRNA–mRNA relationships for further investigation. Future studies incorporating larger cohorts and targeted functional validation will help refine these regulatory relationships and clarify their mechanistic roles in myocardial hypoxia tolerance.

## 4. Materials and Methods

### 4.1. Ethics Statement

All experimental animal procedures were performed in accordance with the China Agricultural University’s regulations for animal care and handling. The animal study protocol was approved by the China Agricultural University Animal Care and Use Committee on 8 February 2023 (permit number: AW80203202-1-1).

### 4.2. Experimental Animals and Sample Collection

This study employed a 2 × 2 factorial design involving two pig breeds (Tibetan and Yorkshire) and two rearing altitudes: high altitude (3000 m; Linzhi, Tibet, China) and low altitude (100 m; Beijing, China). Four breed-by-altitude groups were included: TH, TL, YH, and YL. Within each population, three unrelated six-month-old male pigs of comparable body weight (60–65 kg) were randomly selected (*n* = 3 per group), resulting in 12 animals in total. The individual pig was considered the experimental unit. The YH and TL groups were derived from populations relocated to the corresponding locations approximately three years before sampling and had undergone one generation of local breeding. Animals at both locations received commercial feed of the same brand and formulation. Following electrical stunning and exsanguination, tissue samples were collected immediately from the same region of the right ventricle in all 12 pigs across the four experimental groups, snap-frozen in liquid nitrogen, and stored at −80 °C until RNA extraction. The same 12 heart samples were used for both ribosomal RNA (rRNA)-depleted strand-specific long-RNA library construction and small RNA library construction.

### 4.3. RNA Library Preparation and Sequencing

Total RNA was isolated from heart tissue using TRIzol^®^ Reagent (Ambion, Austin, TX, USA). RNA quality was assessed by 1% agarose gel electrophoresis, and RNA concentration was measured using a NanoDrop™ 2000 spectrophotometer (Thermo Fisher Scientific, Wilmington, DE, USA). RNA integrity was evaluated using an Agilent 2100 Bioanalyzer (Agilent Technologies, Santa Clara, CA, USA). All 12 RNA samples had an RNA integrity number (RIN) ≥ 7.0 and were retained for library construction. One small RNA library was prepared for each animal using 3 μg of total RNA and the NEBNext^®^ Multiplex Small RNA Library Prep Set for Illumina^®^ (New England Biolabs, Ipswich, MA, USA). Briefly, the 3′ SR adaptor was ligated to the 3′ end of the small RNAs. The SR reverse-transcription primer was then hybridized to the 3′ adaptor, followed by ligation of the 5′ SR adaptor. The adaptor-ligated small RNAs were reverse-transcribed into complementary DNA (cDNA) and amplified by polymerase chain reaction (PCR). The resulting cDNA fragments of approximately 140 bp were size-selected by 6% polyacrylamide gel electrophoresis and assessed using the Agilent 2100 Bioanalyzer. The 12 libraries were sequenced by Biomarker Technologies Corporation (Beijing, China) on an Illumina NovaSeq 6000 platform (Illumina, San Diego, CA, USA) to generate 50-bp single-end reads.

### 4.4. miRNA Identification and Expression Analysis

Raw reads were processed to remove adaptor sequences, low-quality reads, reads shorter than 18 nt or longer than 30 nt, and reads containing more than 10% unknown nucleotides. The clean reads were aligned to the Silva, GtRNAdb, Rfam, and Repbase databases using Bowtie (v1.0.0) to remove rRNA, transfer RNA (tRNA), small nucleolar RNA (snoRNA), small nuclear RNA (snRNA), and repetitive sequences. The remaining unannotated reads were aligned to the *Sus scrofa* reference genome (Sscrofa11.1, Ensembl release 103) using Bowtie (v1.0.0) [33]. Known miRNAs were identified by comparison with mature porcine miRNA sequences in miRBase (v22.0) [34], allowing at most one mismatch. Novel miRNAs were predicted using miRDeep2 (v2.0.1.2) [35] based on the secondary structures of their precursor sequences. Putative novel miRNA candidates were retained using a minimum miRDeep2 score of 0, a precursor length of 60–100 nt, Randfold *p* < 0.05, and minimum free energy ≤ −20 kcal/mol. These computational predictions were not considered experimentally confirmed miRNAs. Putative novel miRNA candidates were assigned internal identifiers in the format novel-miR-N, with numeric suffixes ordered according to candidate detection order. Raw miRNA read counts were used for differential-expression and breed-by-altitude interaction analyses in DESeq2. Transcripts per million (TPM) were calculated as (miRNA read count/total clean reads) × 10^6^ and were used for descriptive expression summaries, principal component analysis, heatmaps, and expression-pattern clustering. Nucleotide bias at each miRNA position and small RNA length distributions were also examined to characterize the sequencing data.

### 4.5. Differential Expression and Breed-by-Altitude Interaction Analysis

Differential expression analysis was performed on raw miRNA counts using DESeq2 (v1.22.2) [36]. Four pairwise contrasts were examined: TH vs. YH, TH vs. TL, TL vs. YL, and YH vs. YL. For pairwise comparisons, negative-binomial generalized linear models were fitted with experimental group as the design factor, and differential expression for each contrast was evaluated using the Wald test implemented in DESeq2. Breed-by-altitude interactions were assessed separately using a likelihood-ratio test by comparing the full model (~breed + altitude + breed:altitude) with the reduced model (~breed + altitude). Interaction effects were expressed as (TH − TL) − (YH − YL), with positive values indicating a greater altitude-associated difference in Tibetan pigs than in Yorkshire pigs. For exploratory screening, miRNAs with |log_2_ Fold Change| ≥ 1 and nominal *p* < 0.05 were retained as candidate differentially expressed or interaction-responsive miRNAs. DESeq2 default independent filtering was applied. Hierarchical clustering and UpSetR(v1.4.0) [37] were used to visualize miRNA expression patterns and intersections among comparisons.

### 4.6. K-Means Clustering and Functional Enrichment

The 94 interaction-responsive miRNAs were grouped using k-means clustering. TPM values were averaged within each experimental group, transformed as log_2_ (TPM + 1), and standardized row-wise to Z scores across TH, TL, YH, and YL. Four clusters were specified, with 20 random initializations and a random seed of 123. Candidate target genes of differentially expressed and interaction-responsive miRNAs were predicted using miRanda (v3.3a) [38], with a score ≥ 140 and minimum free energy ≤ −20 kcal/mol. KEGG pathway enrichment analysis of predicted target genes was performed using the R package clusterProfiler (v4.14.6) [39], with *Sus scrofa* specified as the organism. Genes expressed in at least one of the 12 cardiac samples were used as the background. Enrichment was evaluated using a one-sided hypergeometric test, and *p* values were adjusted using the Benjamini–Hochberg procedure. Adjusted *p* < 0.05 was considered statistically significant.

### 4.7. Integrated miRNA–mRNA Network Analysis

Cluster 1 interaction-responsive miRNAs were integrated with downregulated genes identified in the previously published TH vs. YH cardiac transcriptomic comparison [40] to identify candidate miRNA–mRNA regulatory relationships. The transcriptomic data were generated from the corresponding TH and YH heart samples used in the present study. Predicted miRNA–mRNA pairs were retained when their expression levels showed a negative Pearson correlation (r < −0.7, *p* < 0.05). The resulting network was constructed and visualized using Cytoscape (v3.7.2) [41]. Hub miRNAs were identified according to node degree. Functional modules related to cardiac function and structural remodeling, metabolic regulation, and hypoxia sensing and cell survival were defined according to KEGG annotations of the candidate target genes.

### 4.8. qRT-PCR Validation and Correlation Analysis

The expression of three prioritized miRNAs (ssc-miR-671-5p, novel-miR-212, and ssc-miR-296-3p) and their candidate target genes (*ATP1B2*, *ADCY6*, and *PFKM*) was examined by qRT-PCR. The analysis was performed on the same 12 biological samples used for sequencing, following the procedures described previously [40]. Reactions were conducted using a Bio-Rad CFX96 Real-Time PCR System (Bio-Rad Laboratories, Hercules, CA, USA). For mRNA analysis, cDNA was synthesized using the FastKing RT Kit and quantified using a TIANGEN SYBR Green quantitative PCR (qPCR) kit (TIANGEN Biotech, Beijing, China). For miRNA analysis, the miRcute Plus miRNA First-Strand cDNA Kit and miRcute Plus miRNA qPCR Kit were used (TIANGEN Biotech, Beijing, China). Each biological sample was analyzed in three technical replicates. No-template controls were included, and amplification specificity was confirmed by melting-curve analysis. miRNA primers were designed using miRprimer2 [42], whereas mRNA primers were designed using Primer-BLAST (National Center for Biotechnology Information, NCBI) [43]. All primer sequences are provided in Table S1. Relative expression was calculated using the 2^−ΔΔCt^ method [44], with U6 and *HPRT* as reference genes for miRNAs and mRNAs, respectively. Pearson correlations were calculated across the 12 biological samples for the three selected miRNA–mRNA pairs.

### 4.9. Statistical Analysis

Relative expression levels measured by qRT-PCR and calculated using the 2^−ΔΔCt^ method were analyzed by two-way analysis of variance (ANOVA) to test breed, altitude, and their interaction, followed by Tukey’s multiple-comparison test. Data are presented as the mean ± standard error of the mean (SEM), with *p* < 0.05 considered statistically significant.

## 5. Conclusions

In summary, this study demonstrates that breed-by-altitude interaction is associated with distinct cardiac miRNA expression patterns in pigs and identifies a Tibetan high-altitude-associated miRNA module linked to hypoxia signaling, metabolic remodeling, and myocardial homeostasis. Integration with cardiac mRNA expression further prioritized candidate miRNA–mRNA regulatory relationships associated with the Tibetan high-altitude cardiac response. Together, these findings highlight breed-dependent, altitude-associated differences in cardiac miRNA expression and provide molecular candidates for future mechanistic studies of high-altitude adaptation in pigs.

## Data Availability

The sequencing data from the 12 biological samples, including the rRNA-depleted strand-specific long-RNA and small RNA libraries, are publicly available in the NCBI Sequence Read Archive under BioProject PRJNA880668 (https://www.ncbi.nlm.nih.gov/search/all/?term=PRJNA880668; accessed on 7 September 2026).

## Supplementary Materials

The following supporting information can be downloaded at: https://www.mdpi.com/article/10.3390/ijms27209246/s1.

## Abbreviations

The following abbreviations are used in this manuscript:

Abbreviation	Full term
3′ UTR	3′ untranslated region
ANOVA	Analysis of variance
cAMP	Cyclic adenosine monophosphate
cDNA	Complementary DNA
DE	Differentially expressed
FoxO	Forkhead box O
GnRH	Gonadotropin-releasing hormone
HIF	Hypoxia-inducible factor
HIF-1	Hypoxia-inducible factor 1
KEGG	Kyoto Encyclopedia of Genes and Genomes
MFE	Minimum free energy
miRNA	MicroRNA
mRNA	Messenger RNA
mTOR	Mechanistic target of rapamycin
NCBI	National Center for Biotechnology Information
PCA	Principal component analysis
PCR	Polymerase chain reaction
qPCR	Quantitative polymerase chain reaction
qRT-PCR	Quantitative real-time polymerase chain reaction
RIN	RNA integrity number
rRNA	Ribosomal RNA
SEM	Standard error of the mean
snRNA	Small nuclear RNA
snoRNA	Small nucleolar RNA
TH	Tibetan pigs at high altitude
TL	Tibetan pigs at low altitude
TPM	Transcripts per million
tRNA	Transfer RNA
YH	Yorkshire pigs at high altitude
YL	Yorkshire pigs at low altitude
